# Electrically Tunable Meta-Waveplate Enabled by Sb_2_Se_3_-Heterogeneously Integrated Piezoelectric MEMS Mirror

**DOI:** 10.3390/mi17060704

**Published:** 2026-06-08

**Authors:** Jianing Li, Rujun Zhou, Ji Wang, Peishuai Wang, Chenning Tao, Si Luo, Yusheng Zhang, Bin Zhang, Mingwei Tang, Yadong Deng, Zhangwei Yu, Daru Chen

**Affiliations:** 1 College of Physics and Electronic Information Engineering, Zhejiang Normal University, Jinhua 321004, China; li838698018@163.com (J.L.); zhourj@zjnu.edu.cn (R.Z.); wangji@zjnu.edu.cn (J.W.); ps_wang@zjnu.edu.cn (P.W.); taochenning@zjnu.edu.cn (C.T.); luosi@zjnu.edu.cn (S.L.); yszhang@zjnu.edu.cn (Y.Z.); binzhang@zjnu.edu.cn (B.Z.); daru@zjnu.cn (D.C.); 2 Hangzhou Institute of Advanced Studies, Zhejiang Normal University, Hangzhou 311231, China; 3 State Key Laboratory of Extreme Photonics and Instrumentation, Zhejiang Key Laboratory of Autonomous Optoelectronic Perception, College of Optical Science and Engineering, Zhejiang University, Hangzhou 310027, China; tangmw@zju.edu.cn

**Keywords:** metasurface waveplate, tunable, phase-change material Sb_2_Se_3_, piezoelectric MEMS mirror

## Abstract

Metasurfaces have emerged as a powerful platform for subwavelength light manipulation, attracting widespread interest for their potential to replace bulky optical components. However, most metasurfaces are statically designed with fixed functionalities. Here, we demonstrate a high-efficiency tunable meta-waveplate by heterogeneously integrating a phase-change Sb_2_Se_3_ layer with a piezoelectric MEMS mirror. Leveraging the reversible amorphous–crystalline transition of Sb_2_Se_3_, combined with MEMS-enabled nanoscale air gap tuning, the metasurface achieves dynamic switching among zero-, half-, and quarter-waveplate functionalities at the communication wavelength of 1550 nm. The device exhibits stable polarization conversion performance under various rotation angles. Furthermore, we developed a nano-quarter-waveplate library on this platform, which provides extensive phase control over the reflected field and enables programmable beam deflection. This tunable architecture opens new avenues for adaptive photonics with dynamically switchable functionalities.

## 1. Introduction

Polarization, a fundamental property of light characterizing the directional oscillation of its transverse electric field, is independent of frequency, phase, and amplitude [1]. This independence provides an essential degree of freedom for enhanced multiplexing, making precise polarization control critical in nonlinear optics [2], advanced imaging [3], and quantum optics [4]. Conventional birefringent waveplates, however, rely on propagation-length-dependent phase accumulation within anisotropic crystals to generate phase retardation, necessitating macroscopic optical path lengths [5,6]. Such geometric constraints severely limit device miniaturization, directly contradicting integration demands in these fields. Optical metasurfaces—planar arrays of subwavelength resonant nanostructures [7,8,9,10,11,12,13,14,15,16]—offer a disruptive solution. Their ultrathin profile and exceptional ability to precisely manipulate scattered light fields, including polarization, effectively address the stringent scaling requirements of modern photonics. Building on this, metasurface waveplates uniquely combine ultracompact footprints, adaptive polarization transformations, and multifunctional operations [17,18,19,20], enabling applications from polarization-multiplexed imaging [21,22,23] to quantum light sources [24,25].

Despite significant advances, most metasurface waveplates remain fundamentally static—their polarization functionalities permanently encoded in fixed nanostructures, severely limiting adaptability in intelligent photonic systems requiring dynamic polarization control. Current research pursues dynamic tuning via electrical [26,27], optical [28], mechanical [29,30,31], phase-change [32,33,34], and chemical stimuli [35]. Among these approaches, piezoelectric Micro-Electro Mechanical System (MEMS) platforms uniquely achieve sub-millisecond reconfiguration through hybrid plasmonic Fabry-Pérot (FP) cavities formed by integrating metasurfaces on glass with thin-film MEMS mirrors [36,37,38,39,40]. Recent studies have combined materials such as liquid crystals or VO_2_ with mechanically tunable metasurfaces, demonstrating that synergy at the material and structural levels can achieve a performance surpassing that of any single mechanism [41,42]. However, existing single- and bi-layer metasurface [43,44] designs critically overlook the transformative potential of a key innovation: heterogeneously integrating functional multilayer films (e.g., phase-change material Sb_2_Se_3_) directly onto the MEMS mirrors themselves. This co-integration paradigm significantly enhances nanophotonic functionality-density through monolithic consolidation of non-volatile switching, spectral phase engineering, and polarization manipulation within an ultracompact architecture. By enabling simultaneous multi-parameter control and a substantial reduction in reliance on traditional cascaded components, it establishes a new paradigm for electrically tunable meta-waveplates that unify multifunctional integration with sub-wavelength compactness.

In this work, we demonstrate a dual-mechanism tunable MEMS meta-waveplate (TMMW) operating in the near-infrared regime, realized through the heterogeneous integration of Sb_2_Se_3_ into a piezoelectric MEMS mirror. This architecture enables on-demand, dynamic switching among zero-waveplate (ZWP), half-waveplate (HWP), and quarter-waveplate (QWP) functionalities at the communication wavelength of 1550 nm, while maintaining robust polarization conversion performance across different meta-atom rotation angles. Furthermore, we introduce two nano-QWP designs derived from the same platform, which not only enable efficient circular-to-linear polarization conversion but also provide a wide phase control over the reflected field. Leveraging these nano-QWPs, we illustrate how the synergistic combination of resonance [45,46] and geometric phases [19,47] can be harnessed to achieve programmable beam deflection in both co- and cross-polarized channels under circularly polarized (CP) excitation. The proposed heterogeneously integrated MEMS-mirror architecture marks a notable stride toward fully integrated adaptive photonic systems, establishing a versatile platform for high-efficiency, electrically tunable, and multifunctional optical applications.

## 2. Results and Discussion

**Figure 1:**
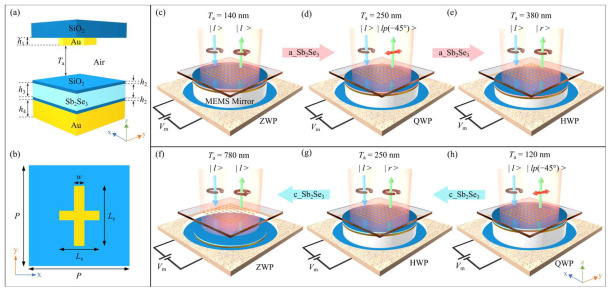
Operating principle of the proposed TMMW in the reflection mode. (**a**) Schematic illustration of a TMMW unit cell. (**b**) Cross-sectional view of the cross-shaped Au meta-atom. (**c**–**e**) With amorphous Sb_2_Se_3_, the TMMW functions as a ZWP with *T*_a_ = 140 nm, a QWP with *T*_a_ = 250 nm, and a HWP with *T*_a_ = 380 nm, respectively. (**f**–**h**) With crystalline Sb_2_Se_3_, the TMMW functions as a ZWP with *T*_a_ = 780 nm, a HWP with *T*_a_ = 250 nm, and a QWP with *T*_a_ = 120 nm, respectively.

Figure 1 illustrates the operating principle of the proposed TMMW device. As depicted in Figure 1a,b, the device adopts a heterogeneously integrated architecture, in which a MEMS mirror coated with an SiO_2_–Sb_2_Se_3_–SiO_2_ multilayer is adhesively bonded to a glass substrate patterned with a metasurface. The MEMS mirror is driven by a lead zirconate titanate (PZT) piezoelectric thin-film actuator integrated beneath the mirror surface. Dynamic modulation is realized through piezoelectric adjustment of the interfacial air gap *T*_a_, controlled by an applied modulation voltage *V*_m_ [36,37,38,39,40]. The phase state of the phase-change material Sb_2_Se_3_ can be reversibly controlled using an electrothermal phase-control scheme. The Joule heating generated by applied electrical pulses induces a phase transition between the crystalline and amorphous states. When Sb_2_Se_3_ is in the amorphous state, progressively increasing *T*_a_ induces sequential polarization transformations in the reflected light. Specifically, incident left-handed circularly polarized (LCP, | *l* >) light first maintains its original polarization state, then to −45° linearly polarized (LP, | *lp*(−45°) >) light, and finally reverts to right-handed circularly polarized (RCP, | *r* >) light. This progression corresponds to the ZWP, QWP, and HWP operational modes, respectively (Figure 1c–e). The analysis takes into account the reversed propagation direction of electromagnetic waves under reflection-mode operation. After switching the Sb_2_Se_3_ thin film to its crystalline state [33,48,49], the TMMW exhibits similar polarization modulation behavior as the cavity length *T*_a_ is progressively reduced (Figure 1f–h). Notably, at a fixed *T*_a_ of 250 nm, on-demand switching between QWP and HWP modes is achieved by reversibly modulating the phase state of the Sb_2_Se_3_ layer (Figure 1d,g).

**Figure 2:**
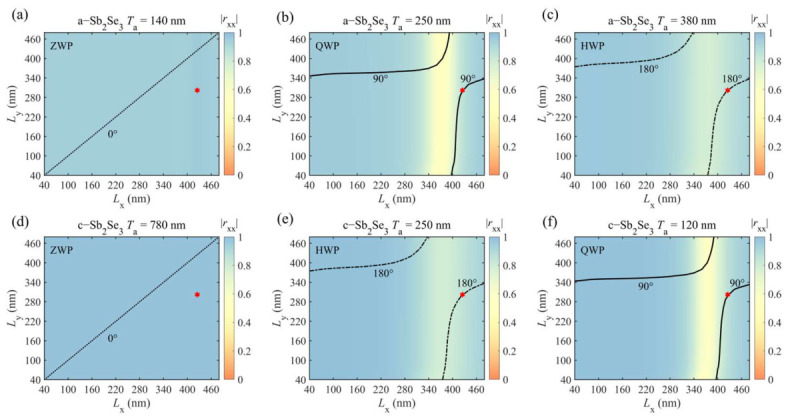
Mapping of the reflection coefficient as a function of meta-atom dimensions under *x*-polarized excitation at the design wavelength of 1550 nm. The color map represents the reflection amplitude $\left|r_{xx}\right|$. Black contours mark the phase difference Δ*δ* = *δ*_yy_ − *δ*_xx_: (**a**,**d**) dotted lines for 0°, (**b**,**f**) solid lines for 90°, and (**c**,**e**) dashed lines for ±180°. Panels (**a**–**c**) and (**d**–**f**) correspond to the amorphous and crystalline states of Sb_2_Se_3_, respectively. The reflection coefficient for *y*-polarization can be obtained by mirroring each map along the line *L*_x_ = *L*_y_.

From a microscopic perspective, the optical reflective response of the proposed TMMW unit-cell, with its principal axes aligned along the *x*- and *y*-directions, can be fully described by its Jones matrix representation:

(1) $$R\;=\;\left(\begin{array}{cc} r_{xx} & 0\\ 0 & r_{yy} \end{array}\right),$$

where the complex reflection coefficients $r_{xx}=\left|r_{xx}\right|e^{i\delta_{xx}}$ and $r_{yy}=\left|r_{yy}\right|e^{i\delta_{yy}}$ correspond to *x*- and *y*-polarized excitations, respectively, with their amplitudes $\left|r_{xx}\right|$ and $\left|r_{yy}\right|$, and phase delays $\delta_{xx}$ and $\delta_{yy}$, primarily determined by the geometric parameters of the cross-shaped gold meta-atom along these orthogonal axes (Figure 1a,b). When the reflection amplitudes are equal (i.e., $\left|r_{xx}\right|=\left|r_{yy}\right|$) and the relative phase difference Δ*δ* = *δ*_yy_ − *δ*_xx_ = ±90° or 180°, the TMMW unit-cell operates ideally as a nano-QWP or a nano-HWP. To ensure high reflection efficiency and provide sufficient resonance phase coverage at the design wavelength of 1550 nm, the unit-cell parameters are set as [*h*_1_, *h*_2_, *h*_3_, *h*_4_, *P*, *w*] = [50 nm, 20 nm, 100 nm, 130 nm, 500 nm, 50 nm]. Three-dimensional (3D) full-wave simulations were conducted using Finite Element Method, with a parametric sweep over the dimensions *L*_x_ and *L*_y_ of the top gold meta-atom to obtain the complex reflection coefficients under *x*- and *y*-polarized excitations. The TMMW unit-cell is illuminated by normally incident *x*- or *y*-polarized plane waves, with periodic boundary conditions applied in both the *x*- and *y*-directions. The periodicity *P* of the TMMW unit-cell is set to 500 nm, which is much smaller than the design wavelength of *λ* = 1550 nm, thereby suppressing higher-order diffraction. A glass domain is introduced above the meta-atom and truncated with a perfectly matched layer to avoid any reflection. The relative permittivity of gold is modeled using the Drude model fitted with experimental data [50], while SiO_2_ is considered as a lossless material with a constant refractive index of 1.46. Moreover, the measured refractive indices of Sb_2_Se_3_ in the amorphous and crystalline states are 3.306 and 4.3281 + 0.000021i, respectively, at the target wavelength of 1550 nm [49]. In the simulations, the wavelength-dependent *n*(*λ*), *k*(*λ*) data from the same reference [49] are used over the 1500–1600 nm range. Figure 2 presents the simulated reflection amplitude and phase distributions of the TMMW unit-cell under *x*-polarized excitation for both amorphous and crystalline states across varying air gap *T*ₐ, with results directly corresponding to the schematic illustrations in Figure 1c–h. Systematic parameter sweeps were performed on the meta-atom dimensions (*L*_x_ and *L*_y_), ranging from 40 nm to 480 nm with a 20 nm step size, while maintaining fixed values for all remaining structural parameters. After parametric optimization, the cross-shaped meta-atom’s final dimensions were established at *L*_x_ = 425 nm and *L*_y_ = 302 nm, as marked by the red hexagon symbols in Figure 2.

**Figure 3:**
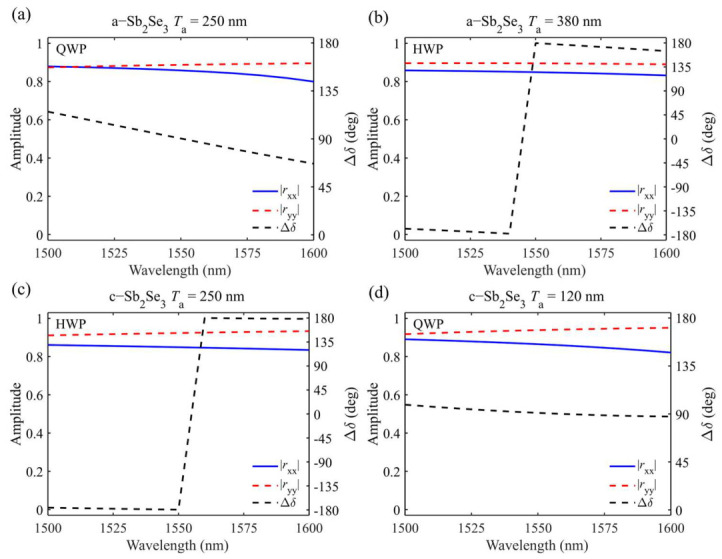
Simulated spectral response of the TMMW under *x*- and *y*-polarized excitations, showing reflection amplitudes ($\left|r_{xx}\right|$ and $\left|r_{yy}\right|$) and relative phase difference (Δ*δ*). Results are given for (**a**) amorphous Sb_2_Se_3_ with *T*_a_ = 250 nm; (**b**) amorphous Sb_2_Se_3_ with *T*_a_ = 380 nm; (**c**) crystalline Sb_2_Se_3_ with *T*_a_ = 250 nm; and (**d**) crystalline Sb_2_Se_3_ with *T*_a_ = 120 nm.

Figure 3 presents the wavelength-dependent reflection amplitudes ($\left|r_{xx}\right|$ and $\left|r_{yy}\right|$) and the relative phase difference (Δ*δ*) under *x*- and *y*-polarized illumination, demonstrating tunable performance across both amorphous and crystalline phases at varying air gap values. At the design wavelength of 1550 nm, the amorphous Sb_2_Se_3_ structure demonstrates reconfigurable waveplate functionality, exhibiting QWP operation with $\left|r_{xx}\right|$ = 0.858, $\left|r_{yy}\right|$ = 0.888, and Δ*δ* = 90.39° at *T*_a_ = 250 nm, and transitioning to HWP operation with $\left|r_{xx}\right|$ = 0.849, $\left|r_{yy}\right|$ = 0.895, and Δ*δ* = 179.66° when *T*_a_ increases to 380 nm (Figure 3a,b). Similarly, in the crystalline state, the structure exhibits HWP functionality with $\left|r_{xx}\right|$ = 0.849, $\left|r_{yy}\right|$ = 0.924, and Δ*δ* = −179.47° at *T*_a_ = 250 nm, and transitions to QWP operation with $\left|r_{xx}\right|$ = 0.865, $\left|r_{yy}\right|$ = 0.939, and Δ*δ* = 91° when *T*_a_ is reduced to 120 nm (Figure 3c,d). Furthermore, $\left|r_{xx}\right|$ and $\left|r_{yy}\right|$ remain consistently above 0.8 within the wavelength range of 1500 to 1600 nm, regardless of the phase state of Sb_2_Se_3_. The relative phase differences Δ*δ* are observed to be very close to ±180° for the HWP and 90° for the QWP. This highlights the functional transition from HWP to QWP for the designed TMMW device, with a broad operating bandwidth of approximately 100 nm centered at 1550 nm.

For further evaluation of the performance of the designed TMMW device, three key polarization parameters of reflected lights are analyzed: the degree of linear polarization (DoLP), degree of circular polarization (DoCP), and angle of linear polarization (AoLP), all derived from Stokes parameters $\left(\begin{array}{c} S_{0}\\ S_{1}\\ S_{2}\\ S_{3} \end{array}\right)\;$ [51]:

(2) $$DoLP\;=\;\sqrt{\left(S_{1}^{2}+S_{2}^{2}\right)}/S_{0},$$

(3) $$DoCP\;={\;S}_{3}/S_{0},$$

(4) $$AoLP\;={\;tan}^{-1}\left(S_{2}/S_{1}\right)/2.$$

**Figure 4:**
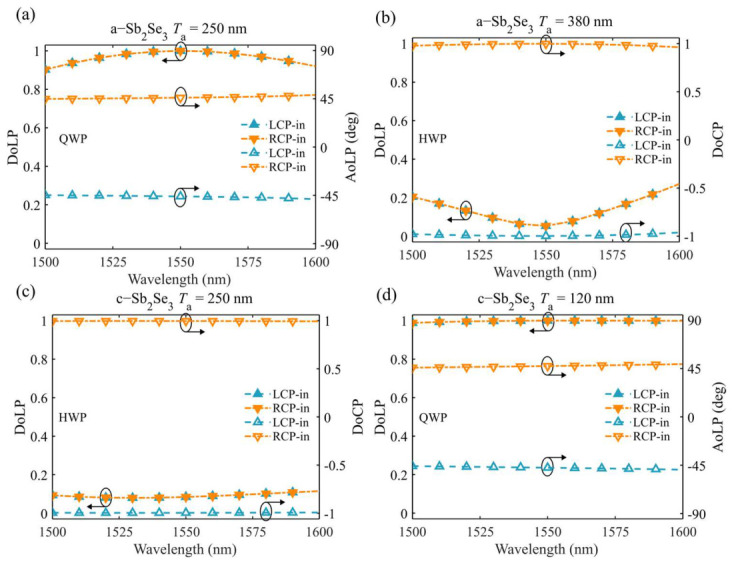
Performance of the TMMW under circularly polarized excitation. (**a**) DoLP and AoLP of the reflected beam for amorphous Sb_2_Se_3_ at *T*_a_ = 250 nm; (**b**) DoLP and DoCP of the reflected beam for amorphous Sb_2_Se_3_ at *T*_a_ = 380 nm; (**c**) DoLP and DoCP of the reflected beam for crystalline Sb_2_Se_3_ at *T*_a_ = 250 nm; (**d**) DoLP and AoLP of the reflected beam for crystalline Sb_2_Se_3_ at *T*_a_ = 120 nm. The short black arrows point to the vertical axis that corresponds to the data set.

Figure 4 presents polarization characteristics of the reflected light under LCP and RCP excitations across the 1500–1600 nm wavelength range, comparing Sb_2_Se_3_ in amorphous and crystalline states under respective air gap configurations. For QWP operation (Figure 4a,d), DoLP and AoLP are shown, whereas for HWP operation (Figure 4b,c), DoLP and DoCP are presented. In the QWP mode-realized with amorphous Sb_2_Se_3_ at *T*_a_ = 250 nm (Figure 4a) or crystalline Sb_2_Se_3_ at *T*_a_ = 120 nm (Figure 4d)—the device efficiently converts incident LCP and RCP light into −45° and +45° linearly polarized light, respectively. The DoLP remains above 0.90 throughout the spectral range, reaching approximately 1 at 1550 nm, indicating high linear polarization purity. In the HWP mode—achieved with amorphous Sb_2_Se_3_ at *T*_a_ = 380 nm (Figure 4b) or crystalline Sb_2_Se_3_ at *T*_a_ = 250 nm (Figure 4c)—the reflected beams exhibit near-ideal circular polarization conversion, with DoCP values approaching −1 and +1 under LCP and RCP excitation, respectively. The corresponding DoLP remains low, below 0.271, over the entire wavelength range.

**Figure 5:**
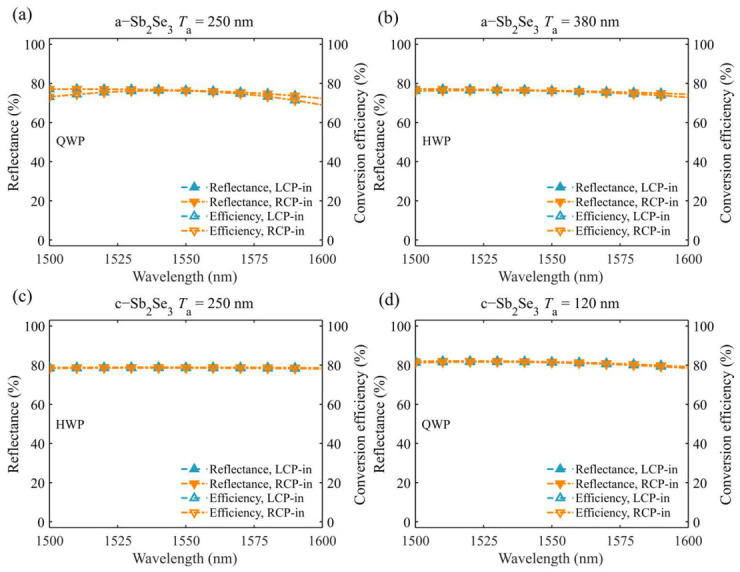
Reflectance and conversion efficiency of the TMMW under the circularly polarized excitation. Results are shown for (**a**) amorphous state with *T*_a_ = 250 nm; (**b**) amorphous state with *T*_a_ = 380 nm; (**c**) crystalline state with *T*_a_ = 250 nm; and (**d**) crystalline state with *T*_a_ = 120 nm.

The optical performance of the TMMW is further quantified in terms of reflectance and conversion efficiency, defined as the intensity ratio of reflected to incident light and the ratio of light converted into the target polarization state to the incident light, respectively. As summarized in Figure 5, under all four operational configurations, both reflectance and conversion efficiency consistently exceed 69.0%, with peak values surpassing 82.1%. At the target wavelength of 1550 nm, the reflectance and conversion efficiency are closely matched, showing a relative deviation of less than 0.4%. Across the entire 1500–1600 nm band, the deviation remains below 4%, underscoring the robust and consistent polarization conversion performance of the TMMW in both HWP and QWP modes.

When the meta-atom satisfying Δ*δ* = 90° is rotated by an angle *θ* with respect to the x-axis, the corresponding Jones matrix is given by:

(5) $$J\left(\theta \right)\;=\;\left|r_{xx}\right|e^{i\delta_{xx}}M^{-1}\left(\theta \right)\left(\begin{array}{cc} 1 & 0\\ 0 & i \end{array}\right)M\left(\theta \right),$$

where $M\left(\theta \right)=\left(\begin{array}{cc} cos\theta & sin\theta \\ -sin\theta & cos\theta \end{array}\right)$ represents the rotation matrix. For the LCP incident light with $E_{in}=\frac{1}{\sqrt{2}}\left(\begin{array}{c} 1\\ i \end{array}\right)$, with AoLP given by *θ* − π/4:

(6) $$E_{out}\;=\;\left|r_{xx}\right|e^{i\delta_{xx}}e^{i\theta }\left(\begin{array}{c} cos\left(\theta -\frac{\pi }{4}\right)\\ sin\left(\theta -\frac{\pi }{4}\right) \end{array}\right),$$

Similarly, for a meta-atom with Δ*δ* = 180°, the Jones matrix becomes:

(7) $$J\left(\theta \right)\;=\;\left|r_{xx}\right|e^{i\delta_{xx}}M^{-1}\left(\theta \right)\left(\begin{array}{cc} 1 & 0\\ 0 & -1 \end{array}\right)M\left(\theta \right).$$

In this case, under LCP incidence, the output is RCP:

(8) $$E_{out}\;=\;\frac{\sqrt{2}}{2}\left|r_{xx}\right|e^{i\delta_{xx}}e^{i2\theta }\left(\begin{array}{c} 1\\ -i \end{array}\right).$$

**Figure 6:**
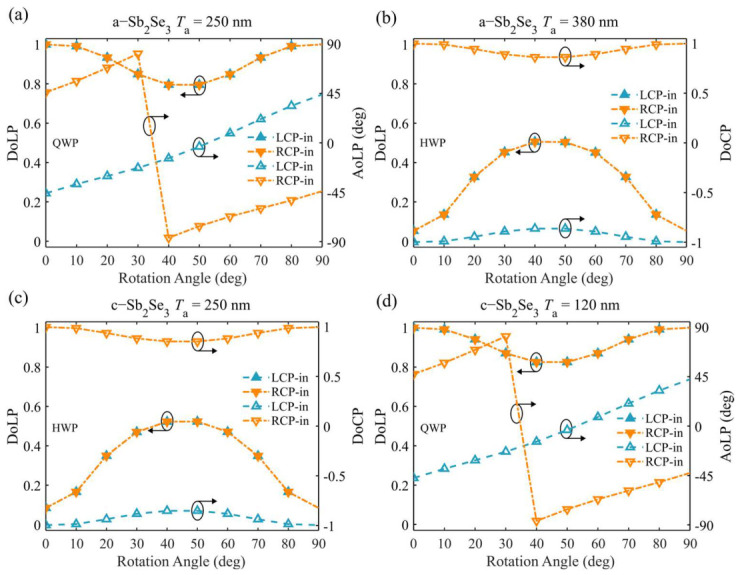
Performance of the TMMW with different rotations relative to the *x*-axis. DoLPs and AoLPs of the reflected beam under CP excitations at the amorphous state with *T*_a_ = 250 nm (**a**) and the crystalline state with *T*_a_ = 120 nm (**d**). DoLPs and DoCPs of the reflected beams under CP excitations at the amorphous state with *T*_a_ = 380 nm (**b**) and the crystalline state with *T*_a_ = 250 nm (**c**). The short black arrows point to the vertical axis that corresponds to the data set.

Figure 6 presents the DoLP and AoLP of the reflected beam from the QWP, and the DoLP and DoCP from the HWP, as functions of the in-plane rotation angle at *λ* = 1550 nm. The AoLP varies linearly with the rotation angle (Figure 6a,d), confirming the orientation-independent behavior of the designed QWP. When operating as an HWP, the DoCP of the reflected beam shows only minor fluctuations, with a minimum around 45°, yet remains above 0.852 across all angles (Figure 6b,c). These results demonstrate that the metasurface maintains stable polarization conversion performance under rotation, highlighting its versatility for implementing complex optical functions.

To achieve synchronous and independent phase modulation of co- and cross-polarized reflected waves in the CP basis, we designed new metasurfaces based on the original structure for beam steering applications. Equation (6) indicates that the LP output acquires an additional phase term *δ*_xx_ + *θ*. At the same time, the LP light can be decomposed into two CP components as follows:

(9) Eout = 12|rxx|ei(δxx+π4)(1i)+12|rxx|ei(δxx−π4)ei2θ(1−i).

As expressed in Equation (9), the reflected field consists of two components: a co-polarized wave retaining the helicity of the incident LCP light, and a cross-polarized wave with the reversed helicity. Their reflection coefficients in the circular basis are defined as: $r_{co}\;=\;\frac{\sqrt{2}}{2}\left|r_{xx}\right|e^{i\left(\delta_{xx}+\frac{\pi }{4}\right)}$ and $r_{cr}\;=\;\frac{\sqrt{2}}{2}\left|r_{xx}\right|e^{i\left(\delta_{xx}-\frac{\pi }{4}+2\theta \right)}$. The co-polarized phase $\delta_{\mathrm{co}}\;={\;\delta }_{xx}+\frac{\pi }{4}$ depends solely on the resonance phase, while the cross-polarized phase $\delta_{\mathrm{cr}}\;={\;\delta }_{xx}-\frac{\pi }{4}+2\theta$ is governed by both the resonance phase and the geometric phase, associated with the meta-atom’s dimensions and orientation, respectively. This enables independent manipulation of the co- and cross-polarized circular polarization channels. To validate independent beam steering in two orthogonal CP channels, we designed two gradient metasurfaces (MS1 and MS2) that spatially separate the reflected CP beams. The phase gradients governing the beam steering are given by:

(10) $$\frac{d\delta_{co}}{dx}\;=\;\frac{\partial \left(\delta_{xx}\left(x,y\right)+\frac{\pi }{4}\right)}{\partial x}\;=\;\frac{\Delta \delta_{xx}}{N\cdot P}\;=\;m_{co}\cdot \frac{2\pi }{\Lambda_{sc}},$$

(11) $$\frac{d\delta_{cr}}{dx}\;=\;\frac{\partial \left(\delta_{xx}\left(x,y\right)-\frac{\pi }{4}+2\cdot \theta \left(x,y\right)\right)}{\partial x}\;=\;\frac{\Delta \delta_{xx}+2\cdot \Delta \theta }{N\cdot P}\;=\;m_{cross}\cdot \frac{2\pi }{\Lambda_{sc}}.$$

Here, *θ*(*x*,*y*) denotes the orientation angle of each meta-atom, Δ*θ* is the relative rotation between adjacent meta-atoms, $\Lambda_{sc}$ represents the total period of one supercell, and *m*_co_ and *m*_cross_ are the diffraction orders for the co- and cross-polarized reflected fields, respectively.

**Figure 7:**
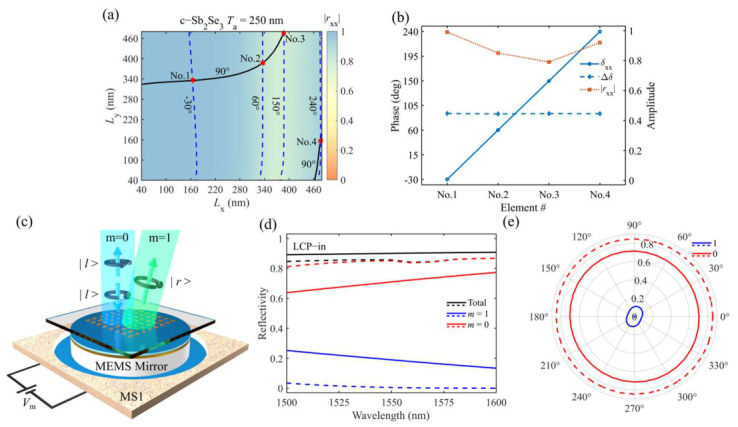
Performance of the designed tunable TMMW MS1. (**a**) Reflection coefficient as a function of meta-atom dimensions under *x*-polarized excitation at 1550 nm for crystalline Sb_2_Se_3_. The color map shows the reflection amplitude |*r*_xx_|, while the blue dashed lines are contours of the reflection phase *δ*_xx_ with a step of 90°, and black solid lines indicate the meta-atoms with the phase difference Δ*δ* equal to 90°. The red hexagonal stars correspond to the four nano-QWPs we selected; (**b**) Calculated reflection amplitude $\left|r_{xx}\right|$, reflection phase *δ*_xx_, and the relative phase difference (Δ*δ* = *δ*_yy_ − *δ*_xx_ = 90°) of four QWP meta-atoms with a 90° resonance phase step at the design wavelength of 1550 nm. The dimensions of elements #1–4 are (1) *L*_X_ = 165 nm, *L*_y_ = 336 nm; (2) *L*_X_ = 336 nm, *L*_y_ = 387 nm; (3) *L*_X_ = 387 nm, *L*_y_ = 476 nm; (4) *L*_X_ = 477 nm, *L*_y_ = 157 nm; (**c**) Schematic of MS1 steering co-polarized (LCP, *m* = 0) and cross-polarized (RCP, *m* = +1) output channels; (**d**) Wavelength-dependent diffraction efficiencies for MS1; (**e**) Polarization state diagrams for the *m* = 0 and *m* = +1 diffracted orders under LCP incidence at *λ* = 1550 nm. The solid line represents the case where Sb_2_Se_3_ is in the crystalline state with a *T*_a_ of 250 nm, while the dashed line represents the case where Sb_2_Se_3_ is in the amorphous state but with *T*_a_ remaining unchanged (**d**,**e**).

As a representative case, using the model parameters derived from Figure 2e (with Sb_2_Se_3_ in the crystalline state and *T*_a_ of 250 nm), we performed a parametric sweep of *L*_x_ and *L*_y_ of the gold cross structures. This analysis yielded the calculated reflection coefficient as a function of the nano-antenna dimensions at the design wavelength of 1550 nm under *x*-polarized illumination. As shown in Figure 7a, the contours corresponding to a phase difference of Δ*δ* = 90° are marked with black solid lines, identifying candidate structures for nano-QWPs. Furthermore, the achievable resonance phase of these nano-QWPs spans a wide range of up to 270°. To ensure broad operational bandwidth and high conversion efficiency, we selected four nano-QWPs (marked by hexagonal stars) exhibiting a phase step of Δ*δ*_xx_ = 90° to constitute a meta-atom library. Figure 7b shows the reflection amplitudes and phases of four selected nano-QWPs (element #1 to #4) with a resonance phase step of Δ*δ*_xx_ = 90° at 1550 nm, which ensure simultaneous circular-to-linear polarization conversion and potential wavefront shaping.

For MS1, LCP incident light is directed into the co-polarized (LCP, *m* = 0) and cross-polarized (RCP, *m* = +1) diffraction channels (with Sb_2_Se_3_ in the crystalline state and *T*_a_ of 250 nm), as illustrated in Figure 7c. Setting *m*_co_ = 0, *m*_cross_ = 1, and *N* = 2 yields the solutions Δ*δ*_xx_ = 0° and Δ*θ* = π/4, realized using an 8-element supercell. We selected Meta-atom No.1 from the nano-QWP library due to its high reflection efficiency and arranged the supercell with orientation angles *θ*(*x*,*y*) of 90°, 90°, 135°, 135°, 180°, 180°, 225°, and 225°, respectively. The elements are spaced at a period of *P* = 500 nm (Figure 7c). As shown solid lines in Figure 7d, nearly all LCP incident light is diffracted into the 0th and +1st orders, with the −1st order strongly suppressed—attributed to the broadband nature of the geometric phase. The simulated diffraction efficiencies reach ~71% and ~19% for the 0th and +1st orders, respectively, at 1550 nm. This confirms the effective spatial separation of orthogonal CP channels. The polarization states of the diffracted beams, analyzed as a function of analyzer angle (solid lines in Figure 7e), exhibit nearly ideal circular distributions, further verifying polarization purity in both output channels. Conversely, when Sb_2_Se_3_ is switched to the amorphous state (with *T*_a_ unchanged), the predefined beam steering functionality is no longer preserved, thereby demonstrating the potential of our TMMW design for information encryption and decryption (dash lines in Figure 7d,e).

**Figure 8:**
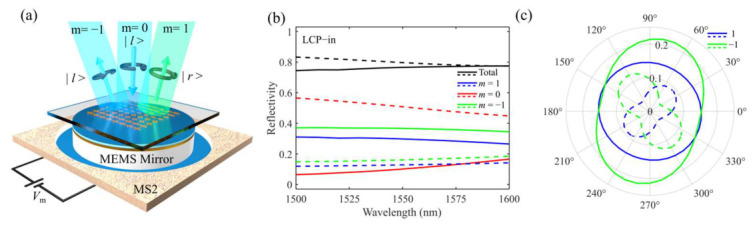
Performance of the designed tunable TMMW MS2. (**a**) Schematic of MS2 (crystalline Sb_2_Se_3_, *T*_a_ = 250 nm) for steering co- and cross-polarized circularly polarized waves into the −1 and +1 diffraction orders, respectively; (**b**) Simulated diffraction efficiencies of different orders as a function of wavelength; (**c**) Polarization state diagrams of the diffracted beams in the −1 and +1 orders under LCP illumination at 1550 nm. The solid line represents the case where Sb_2_Se_3_ is in the crystalline state with a *T*_a_ of 250 nm, while the dashed line represents the case where it is in the same crystalline state but with *T*_a_ changed to 500 nm (**b**,**c**).

By synergizing resonant and geometric phases, MS2 (crystalline Sb_2_Se_3_, *T*_a_ = 250 nm) is designed to direct co- and cross-polarized waves into the −1st and +1st diffraction orders, respectively. Substituting *m*_co_ = −1, *m*_cross_ = 1, and *N* = 2 into Equations (10) and (11) leads to an 8-element supercell with the meta-atom sequence [No.3, No.3, No.2, No.2, No.1, No.1, No.4, No.4] and rotation angles [0°, 0°, 90°, 90°, 180°, 180°, 270°, 270°] (Figure 8a). The simulated results (solid lines, Figure 8b) show that most incident LCP light is diffracted into the target orders, with efficiencies of ~37% (−1st) and ~30% (+1st) at 1550 nm. Furthermore, the nearly circular polarization distributions of these orders (Figure 8c, solid lines) verify successful polarization control in spatially separated channels. Notably, this beam steering functionality is also disrupted when *T*_a_ is switched to 500 nm, even with Sb_2_Se_3_ remaining crystalline.

## 3. Conclusions

In summary, we have realized a TMMW device for the near-infrared by heterogeneously integrating a phase-change Sb_2_Se_3_ film with a piezoelectric MEMS mirror. This platform enables dynamic and reversible switching between HWP and QWP operations at 1550 nm, with robust polarization conversion performance maintained over a wide range of rotation angles. Furthermore, we established a nano-QWP library supporting a resonance phase shift exceeding 270°, which facilitates efficient circular-to-linear polarization conversion alongside extensive phase manipulation of the reflected wavefront. By strategically combining this resonant phase with the geometric phase, we demonstrated metasurfaces that achieve programmable beam deflection in both co- and cross-polarized channels under circular polarization. This heterogeneously integrated architecture represents a significant advancement for adaptive meta-optics, establishing a versatile pathway toward dynamically reconfigurable and highly compact photonic systems.

## Data Availability

The original contributions presented in this study are included in the article. Further inquiries can be directed to the corresponding authors.
